# Sema6A and Mical1 control cell growth and survival of BRAF^V600E^ human melanoma cells

**DOI:** 10.18632/oncotarget.2995

**Published:** 2014-12-18

**Authors:** Rossella Loria, Giulia Bon, Valentina Perotti, Enzo Gallo, Ilaria Bersani, Paola Baldassari, Manuela Porru, Carlo Leonetti, Selene Di Carlo, Paolo Visca, Maria Felice Brizzi, Andrea Anichini, Roberta Mortarini, Rita Falcioni

**Affiliations:** ^1^ Department of Experimental Oncology, Regina Elena National Cancer Institute, Rome, Italy; ^2^ Human Tumors Immunobiology Unit, Dept. of Experimental Oncology and Molecular Medicine, Fondazione IRCCS Istituto Nazionale dei Tumori, Milan, Italy; ^3^ Department of Pathology, Regina Elena National Cancer Institute, Rome, Italy; ^4^ Department of Chemotherapy, Regina Elena National Cancer Institute, Rome, Italy; ^5^ Department of Medical Sciences, University of Turin, Turin, Italy

**Keywords:** BRAF^V600E^ melanoma, NRAS^Q61R^ melanoma, Sema6A, Mical1, cell survival

## Abstract

We used whole genome microarray analysis to identify potential candidate genes with differential expression in BRAF^V600E^ *vs* NRAS^Q61R^ melanoma cells. We selected, for comparison, a peculiar model based on melanoma clones, isolated from a single tumor characterized by mutually exclusive expression of BRAF^V600E^ and NRAS^Q61R^ in different cells. This effort led us to identify two genes, SEMA6A and MICAL1, highly expressed in BRAF-mutant *vs* NRAS-mutant clones. Real-time PCR, Western blot and immunohistochemistry confirmed preferential expression of Sema6A and Mical1 in BRAF^V600E^ melanoma. Sema6A is a member of the semaphorin family, and it complexes with the plexins to regulate actin cytoskeleton, motility and cell proliferation. Silencing of Sema6A in BRAF-mutant cells caused cytoskeletal remodeling, and loss of stress fibers, that in turn induced cell death. Furthermore, Sema6A depletion caused loss of anchorage-independent growth, inhibition of chemotaxis and invasion. Forced Sema6A overexpression, in NRAS^Q61R^ clones, induced anchorage-independent growth, and a significant increase of invasiveness. Mical1, that links Sema/PlexinA signaling, is also a negative regulator of apoptosis. Indeed, Mical-1 depletion in BRAF mutant cells restored MST-1-dependent NDR phosphorylation and promoted a rapid and massive NDR-dependent apoptosis. Overall, our data suggest that Sema6A and Mical1 may represent new potential therapeutic targets in BRAF^V600E^ melanoma.

## INTRODUCTION

The development of mutant BRAF and of MEK1/2 inhibitors has markedly improved treatment of advanced BRAF-mutant melanoma [1-2] showing highly significant effects on progression-free and/or overall survival in several phase III trials, in comparison to chemotherapy [3]. Nevertheless, a fraction of patients does not benefit from target-specific therapy and duration of clinical responses can be limited. Intrinsic and acquired resistance to small molecule inhibitors limit the efficacy of these therapeutic approaches [4-5], highlighting the urgent need for identification of new molecules and pathways that may contribute to melanoma growth. Several screening strategies have been proposed to identify genes with a significant role in BRAF^V600E^melanoma persistence. These include identification of BRAF target genes by comparing gene expression profiles in melanomas treated with/without BRAF inhibitors [7], screening for expression of relevant molecules to be targeted by inhibitors [8-11], and comparison of gene expression profiles of BRAF^V600E^ vs wild-type tumors [12-13]. The latter approach has the advantage of contributing to define the gene expression landscape associated with a frequently activated oncogene, but conflicting results have been published [14-19] possibly due to strong inter-tumor genetic heterogeneity. To reduce the impact of this heterogeneity, we exploited a set of melanoma clones, isolated from a metastasis containing both BRAF^V600E^ and NRAS^Q61R^ cells [20]. The clones, previously shown to have mutually exclusive expression of activated BRAF^V600E^ or NRAS^Q61R^ [20], were used for whole genome gene expression profiling aimed at identifying genes overexpressed in BRAF^V600E^ cells. This approach led us to identify the semaphorin SEMA6A, a ligand of Plexin A2 and A4 [21-23], as one of the most discriminating genes, overexpressed in BRAF-mutant clones compared to NRAS^Q61R^ clones. Interestingly, another gene in the semaphorin-plexin pathway, MICAL1 (molecule interacting with CasL), that links Sema/PlexinA signaling to F-actin disassembly [24-26], showed similar differential expression in BRAF-mutants compared to NRAS-mutant clones. Although the sema/plexin/mical pathway has been thoroughly investigated for its role in repulsive guidance, recent studies point to a role of its different components in regulating cell survival and migratory functions even in neoplastic cells [27]. It has been demonstrated that semaphorine 3A regulates tumor persistence by suppressing apoptosis triggered by plexin D1 dependent receptor [28]. Among this class of molecules, Sema6A regulates vascular development in tumors and angiogenesis by modulating VEGFR2 signaling in endothelial cells [22]. Mical1 is a multi-domain flavoprotein monoxigenase binding NDR1/2 kinases, thus inhibiting both their phosphorylation by MST1 and NDR-mediated apoptosis [26, 29]. We tested whether Sema6A and Mical1 could exert a significant pro-tumoral role in BRAF^V600E^ melanomas by contributing to regulate their growth, survival, and invasion. The results confirmed this hypothesis and suggested that different components of the sema/plexin/mical signaling cascade may represent new therapeutic targets in melanoma.

## RESULTS

### BRAF^V600E^ and NRAS^Q61R^ clones isolated from the same metastatic melanoma have distinct biological behavior and gene expression profiles

**Figure 1:**
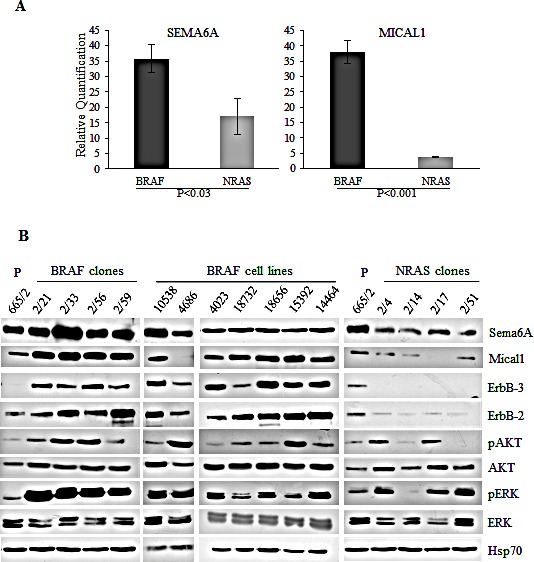
Sema6A and Mical1 are highly expressed in BRAF^V600E^ compared to NRAS^Q61R^ melanoma (A) Average expression of SEMA6A and MICAL1 mRNA examined by qRT-PCR±SD. (B) Total cell lysates from cell line 665/2, from BRAF and NRAS clones isolated from 665/2 and from seven BRAF-mutant cell lines were analyzed by WB for expression of Sema6A, Mical1, ErbB3, ErbB2, total and pAkt, total and pERK, and Hsp70.

The distinct genetic makeup of BRAF-mutant and NRAS-mutant clones derived from the 665/2 cell line [20] was associated with different biological behavior *in vivo*. Upon intracardiac injection, BRAF^V600E^ clones metastatized in all mice and all organs, except for spleen and testis, while only one NRAS^Q61R^ clone was able to metastasize, and only in one mouse and exclusively to the intestine and stomach (Table S1). These marked differences were reflected in the gene expression profiles. Whole genome microarray analysis of BRAF^V600E^ and NRAS^Q61R^ clones (Fig. S1) identified SEMA6A as one of the most discriminating genes, highly expressed in the BRAF^V600E^ clones compared to the NRAS^Q61R^ ones. Another gene belonging to the sema/plexin signaling pathway, MICAL1, was also expressed at significantly higher levels in BRAF clones (Fig. S1). Higher expression of Sema6A and Mical1, at both RNA and protein levels, in BRAF^V600E^ clones, was confirmed by qRT-PCR and WB, respectively (Fig. 1A, and B). We also found high expression of both Sema6A and Mical1 in BRAF^V600E^ cells derived from seven different melanoma cell lines isolated from primary tumor or from lymph node metastases (Fig. 1B). Most BRAF^V600E^ clones and cell lines in this panel showed constitutive activation of the AKT and ERK pathway, while NRAS^Q61R^ clones showed reduced activation of AKT pathway, a finding consistent with the lower/missing expression of ErbB2/ErbB3 receptor, the strongest activator in nature of PI3K [30].

### Sema6A and Mical-1 are highly expressed *in vivo* in BRAF^V600E^ tumors compared to wild type melanomas and nevi

**Figure 2:**
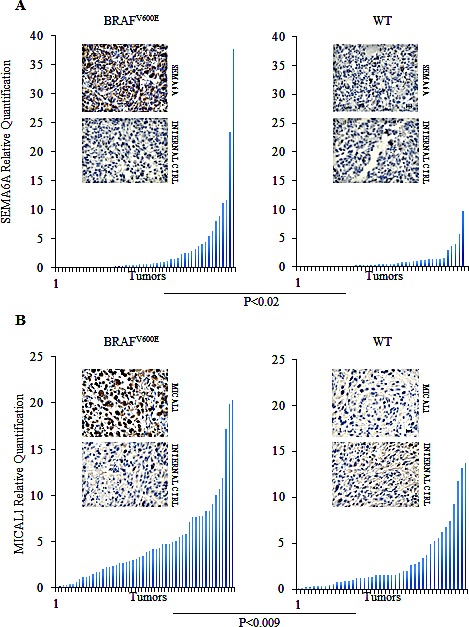
Sema6A and Mical1 are highly expressed *in vivo* in B-RAF^V600E^ compared to WT melanomas (A-B) Expression of SEMA6A and MICAL1 mRNA was examined by qRT-PCR on lymph node biopsies from patients carrying BRAF^V600E^ or WT tumors (left and right graphs). Representative Sema6A and Mical1 IHC on sections derived from same samples. Secondary antibody was used as internal control. Scale bar 30 μm.

To assess whether Sema6A and Mical-1 were preferentially expressed in BRAF^V600E^ tumors, we analyzed their expression by qRT-PCR and immunohistochemistry (IHC) on BRAF^V600E^ and BRAF wild type (WT) melanoma specimens derived from patients surgically treated at the Regina Elena National Cancer Institute. Both molecules were also analyzed in nevi. The results demonstrated that Sema6A and Mical-1 were significantly more expressed in BRAF^V600E^ than in WT melanomas (P<0.02 and P<0.009, respectively) (Fig. 2A and B). In nevi, Sema6A and Mical-1 expression was comparable to the levels found in WT melanoma (Fig. S2). Representative IHC analyses with the relative internal controls are reported for both molecules in the two subtypes of melanoma (Fig. 2A and B) and in nevi (Fig.S2). These data supported our *in vitro* results.

### Depletion of Sema6A in BRAF-mutant melanoma cells promotes cell death

**Figure 3:**
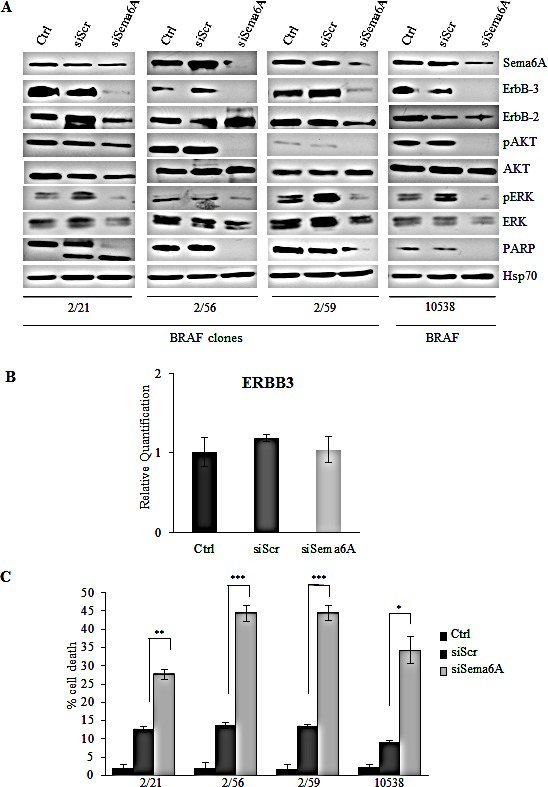
Interference for Sema6A induces cells death, and inhibits PI3K pathway (A) siSema6A/BRAF^V600E^ clones and primary melanoma cell line 10538 were analyzed by WB for expression of Sema6A, ErbB-3, ErbB-2, P-AKT, P-ERK1/2, PARP, and Hsp70. (B) Average expression of ErbB-3 mRNA examined by qRT-PCR±SD in Control, siScr, and siSema6A in clone 2/59. (C) Cell death of siSema6A/BRAF^V600E^ cells was evaluated at 48 h post-transfection by Trypan blue exclusion from three independent experiments; bars ± S.D.

To investigate the role of Sema6A in BRAF^V600E^ cells, we carried out siRNA experiments, with different Sema6A-specific silencing sequences (siSema6A) in three clones isolated from 665/2 cell line and in one cell line (10538) isolated from a BRAF^V600E^ primary tumor. Sema6A depletion strongly induced PARP cleavage in clone 2/21 and reduced total PARP in the clones 2/56 and 2/59 and in the melanoma cell line 10538 (Fig. 3A). Surprisingly, Sema6A silencing inhibited ErbB3 expression and phosphorylation of AKT, and to a lesser extent, of ERK (Fig. 3A). ErbB3 down-regulation was likely post-transcriptional, secondary to p-Akt inhibition, as previously described [31], and not transcriptional, as demonstrated by qRT-PCR (Fig. 3B). The above results were confirmed by silencing of Sema6A by a second specific siSema6A sequence (Fig. S3A), and even by a third specific commercially available siSema6A (data not shown). Interestingly, PI3K and MAPK activity appeared to be regulated downstream of Sema6A, as their phosphorylation levels (Fig. 3A, and S3A) clearly correlated with Sema6A depletion, suggesting that this semaphorin can regulate major pathways supporting melanoma cell viability. To further explore this possibility, we carried out apoptosis assays by FACS analysis. Annexin-V/PI staining assays indicated that Sema6A depletion induced apoptosis, associated with caspase 3/7 activation, in BRAF^V600E^ clones 2/56 and 2/59 and in melanoma cell line 10538, with effects already evident at 24-36h (Fig. S4). Trypan blue exclusion assays confirmed that siSema6A induced death in all cells tested compared to controls or siScr transfected cells (Fig. 3C). Taken together these results suggested that Sema6A promotes survival of BRAF-mutant melanoma cells.

### Depletion of Sema6A in BRAF-mutant melanoma cells alters the cytoskeleton and impairs anchorage-independent growth, as well as motility and invasive activities

**Figure 4:**
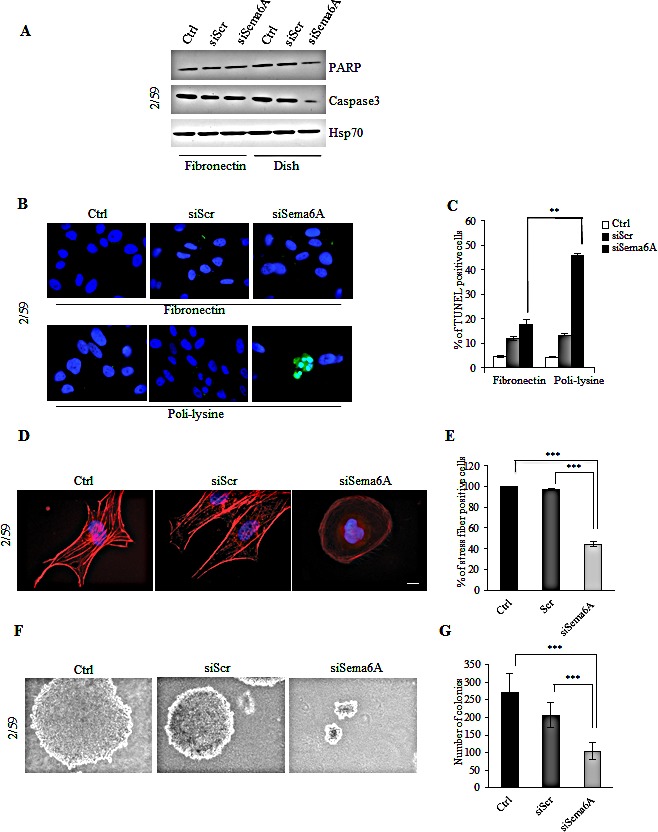
Interference with Sema6A expression in BRAF 2/59 cells induces cytoskeletal remodeling and inhibits anchorage-independent growth (A) Control, siScr- or siSema6A/BRAF^V600E^ cells were plated onto FN coated-dish or conventional culture-dish. Total cell lysates were analyzed by WB with PARP, Caspase3, and Hsp70 antibodies. (B) Tunel assay of BRAF/siSema6A cells plated on FN and/or poli-lysine. (C) Histogram reported percentage of tunel positive cells (P<0.004). (D) The cells, 48 h post-transfection, were plated on poly-l lysine coated slides, and stained with phalloidin-TRITC to show actin filaments. Scale bar is 10 μm. (E) Histogram reported percentage of stress fiber positive-cells. (F) Ctrl, siScr and siSema6A/BRAF^V600E^ cells soft agar assay. (G) Histogram reported the number of colonies obtained in soft agar assay in Ctrl, siScr and siSema6A cells.

Sema6A silencing led to down-regulation of Caspase 3 and reduction of total PARP when cells where plated on conventional cell culture dishes, but not on Fibronectin-coated dishes suggesting cell death by loss of cell adhesion (Fig. 4A). The Cell death was confirmed by TUNEL assays showing fragmentation of nuclei only in siSema6A cells plated on poli-lysine (Fig. 4B). Graphical representation of percentage of TUNEL positive cells is reported in Fig. 4C (P<0.004). Silencing of Sema6A also induced changes in the actin cytoskeleton as shown by loss of actin stress fibers consistent with impaired cell adhesion and with promotion of cell death by anoikis (Fig. 4D, and E, P<0.0001). These results were further confirmed in clones 2/56, 2/21, and in 10538 cell line (Fig. S5A and B, right panels). These changes were not observed in untreated or siScr/cells (Fig. 4D, and E, and Fig. S5A and B, left and central panels). These results suggested that Sema6A might contribute to anchorage-independent growth of BRAF^V600E^ melanoma cells. Indeed, Sema6A depletion strongly suppressed growth of BRAF^V600E^ cells in soft agar (Fig. 4F, and G). siSema6A cells formed very small colonies appearing later, compared to control or to siScr/cells (P<0.0001) confirming that Sema6A controls anchorage-independent growth. In agreement with above results, Sema6A depletion in BRAF mutant cells strongly reduced their capability to migrate and to invade *in vitro* (P≤0.001) (Fig. S6A and B, upper and lower panels).

### Ectopic expression of Sema6A in NRAS-mutant cells promotes *in vitro* and *in vivo* invasion

**Figure 5:**
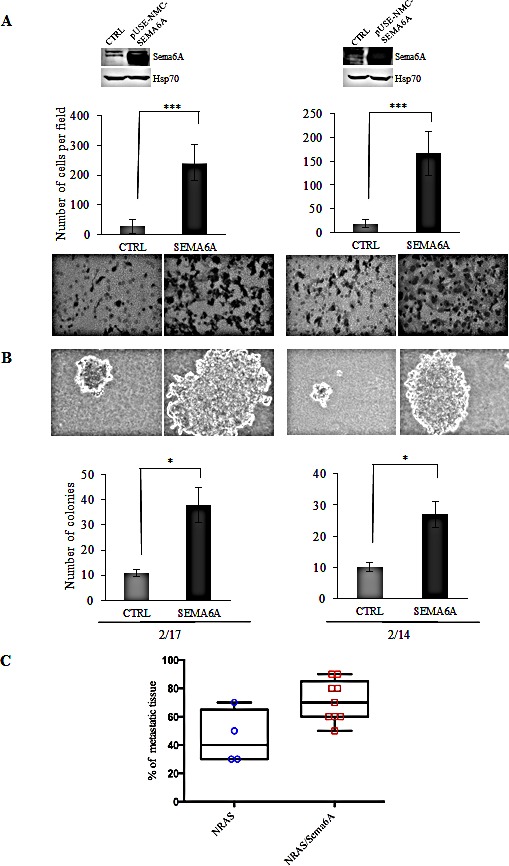
Sema6A controls invasion (A) Overexpression of Sema6A by WB (upper part of the figure), and invasion assay (lower part of the figure), and (B) soft agar assay, of control and Sema6A/NRAS^Q61R^ overexpressing clones (2/14 and 2/17). (C) Box plot of *in vivo* skeletal tissues metastases analysis was obtained by software Prism version 6.0.

We then tested whether forced overexpression of Sema6A in NRAS^Q61R^ clones could promote anchorage-independent growth and invasiveness. In contrast with *in vivo* results on development of metastases (Table S1) and with data indicating that NRAS mutant clones yield only small colonies in soft agar [20], we found that overexpression of Sema6A in NRAS^Q61R^ melanoma clones conferred a strong invasive activity *in vitro* (P≤0.0001) (Fig. 5A), and capability to generate large colonies in soft agar (P≤0.02) (Fig. 5B). Furthermore, depletion of Sema6A in NRAS^Q61R^ melanoma clones down-regulated their capability to invade (P≤0.001) (Fig. S7). More importantly, by *in vivo* assessment for metastatic ability of NRAS-mutant cells overexpressing Sema6A, we found that several components of the skeletal system of the mice (spinal column, femurs, and upper and lower limbs) were characterized by presence of metastatic foci. Moreover, we found a significant increase, at these anatomical sites, in the fraction of tissue being interested by metastatic foci, compared to tissues from mice receiving control NRAS-mutant cells (Fig. 5C). Analysis of additional organs (Table S2) demonstrated that NRAS cells overexpresing Sema6A frequently produced metastases also in the brain, lung, pancreas and heart, compared to control cells. These data strongly support the role of Sema6A in the mechanisms that promote melanoma invasion.

### Sema6A functions in BRAF-mutant melanoma cells are independent from PlexinA2 and PlexinA4 receptors

We then looked at expression and function in melanoma cells of Plexin-A2 and Plexin-A4, the Sema6A receptors. In agreement with previous results [32], all BRAF^V600E^ clones and cell lines expressed heterogeneous levels of Plexin-A4 (Fig. S8A, and B) compared to the level found for Sema6A (Fig. 1), while all clones and cell lines were negative for the expression of Plexin-A2 (data not shown). Both PlexinA4 and PlexinA2 were absent in NRAS^Q61R^ clones (Fig. S8A and B, and data not shown). Surprisingly, depletion of Plexin-A4 in BRAF^V600E^ clone by siRNA did not induce cell death and did not alter the actin cytoskeleton (data not shown). Silencing of both molecules led to the expected changes already found upon siSema6A alone (Fig. S8C). Taken together these results suggest that Sema6A and Plexin-A4 have distinct roles in regulating biological functions in BRAF-mutant melanoma cells.

### Mical1 depletion induces melanoma cell death by apoptosis

**Figure 6:**
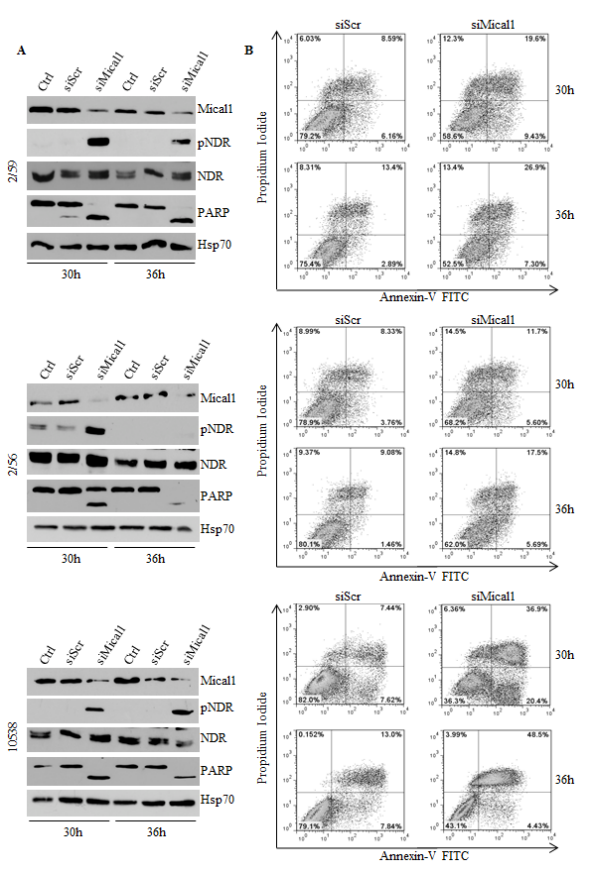
Silencing Mical1 induces apoptosis (A) siMical-1/BRAF^V600E^ cell lysates from 2/59 and 2/56 clones, and 10538 primary tumor were analyzed at the indicated time post-transfection for expression of Mical1, total and pNDR, and PARP. (B) Apoptosis was evaluated by Annexin-V/PI flow cytometric assay.

**Figure 7:**
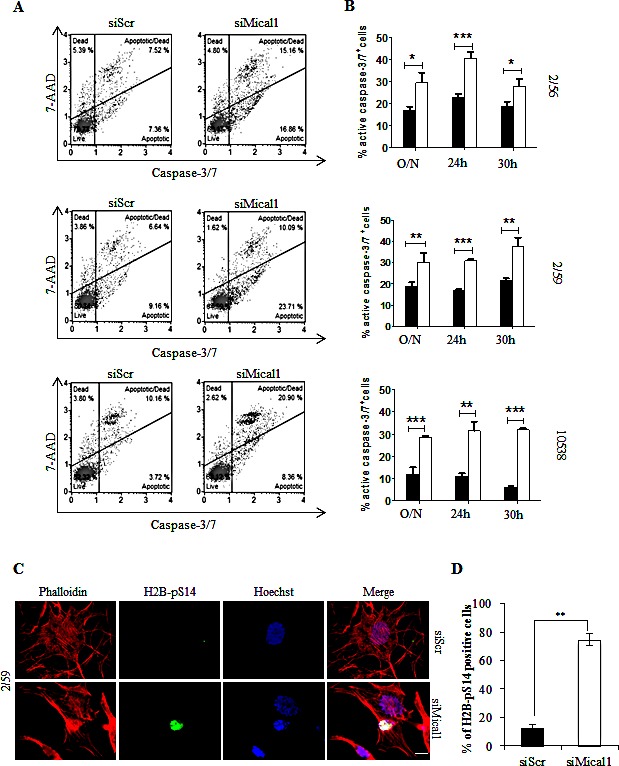
Silencing Mical1 induces Caspase 3/7 activation and H2B phosphorylation on Serine 14 (A) siMical-1/BRAF^V600E^cells were analyzed with Muse™ Caspase-3/7 assay. (B) Caspase-3/7 activity after transfection with siScr (black bars) or siMICAL1 (white bars) is reported. (C) Cells plated on poly-l lysine coated slides, stained with phalloidin-TRITC, and anti-H2B-pS14 that co-stains with Hoechst of apoptotic nuclei of siMical-1/BRAF^V600E^ cells. (D) Scale bar is 10 μm. Histogram reported the percentage of H2B-pS14 positive cells.

Mical1 is a mediator of Sema/Plexin repulsion, and acts by promoting F-actin disassembly [33-34]. Mical1 enhanced expression in BRAF^V600E^ clones, and its known role as an effector of the Sema/Plexin axis, suggested that it could exert functions similar to those regulated by Sema6A. To address this possibility we depleted Mical1 by siRNA. Surprisingly, Mical1 silencing did not change the actin cytoskeleton organization, indicating that its role in BRAF^V600E^ melanoma cells is not overlapping with that of Sema6A. A candidate function for Mical1 is interaction with NDR, a pro-apoptotic kinase, antagonizing MST1-induced NDR phosphorylation to trigger apoptosis [29]. Indeed, silencing of Mical1 in BRAF^V600E^ melanoma clones (2/59, 2/56) and in 10538 melanoma cell line caused strong NDR phosphorylation between 30 h and 36 h post transfection associated with PARP cleavage (Fig. 6A). By Annexin-V/PI flow cytometry assay, we also found that Mical1 depletion in these melanoma cells increased both early (annexin-V^+^PI^−^) and late (Annexin-V^+^PI^+^) apoptosis, compared with the effects seen in cells transfected with control siRNA (siScr, Fig. 6B), suggesting that Mical-1 protects BRAF^V600E^ melanomas from apoptosis. These results were confirmed by the use of a second siMical1 (Fig. S3B), and by a commercially available siMical1 (data not shown). By caspase-3 activation assays on the same cells, we confirmed that siMical1 induced melanoma apoptosis (Fig. 7A, B). Analysis for phosphorylation of H2B-S14, that co-localizes with Hoechst positivity in fragmented nuclei, further supported the evidence that siMical1 induces apoptosis in BRAF^V600E^ melanoma without affecting the actin cytoskeleton (Fig. 7C, D). Taken together, these results support the notion that both Sema6A and Mical1 promote cell survival, and inhibit cell death of BRAF^V600E^ melanomas through different pathways.

## DISCUSSION

In attempts to find molecules regulating aggressiveness of BRAF^V600E^ melanoma cells, by whole-genome array we found that SEMA6A and MICAL1, two genes of the SEMA/PLEXIN signaling pathway, were over-expressed in BRAF^V600E^ clones compared to NRAS^Q61R^ clones isolated from the same metastasis. *In vitro* and *in vivo* assays confirmed the selective expression of the corresponding proteins in BRAF^V600E^ cell lines derived from primary and metastatic melanoma lesions or in BRAF mutant tumors compared to BRAF wild type tumors and nevi. Sema6A belongs to a large family of secreted or trans-membrane proteins and its receptors are PlexinA2 and PlexinA4. Sema6A/Plexin complexes were previously characterized for their role in vascular and neural development [22]. However a role of SEMA6A in cell proliferation, survival, anchorage-independent cell growth, and invasion of BRAF^V600E^ melanoma was not previously described. We found that Sema6A-depletion in BRAF^V600E^ clones and primary tumor cells results in cell death. Specifically, Sema6A depletion reduces Akt phosphorylation and suppresses ErbB-3 expression suggesting that its inhibition of cell proliferation could depend on loss of ErbB-2/ErbB-3 heterodimer formation. Loss of Sema6A by siRNA caused a very low reduction of ErbB-2 expression and of ERK activity suggesting that in these cells ErbB-2 homodimer may still contribute to cell proliferation. However, when the cells were forced to adhere to fibronectin (FN) we did not find cleavage of PARP and activation of caspase 3.

Previous studies have shown that depletion of Sema6A in endothelial cells reduces VEGFR2 expression and abolish its phosphorylation, thus inducing cell death. Interestingly, stimulation by VEGF did not restore cell proliferation in Sema6A-depleted endothelial cells, demonstrating that Sema6A mainly control proliferation through the VEFGR2 receptor [22]. Overall, the results of this study add further evidence for a direct role of SEMA6A as regulator of cell proliferation even in neoplastic cells.

Cells with depleted Sema6A showed loss of stress fiber and actin distribution exclusively under the cell membrane. This was associated with loss of adhesion to the substrate, reduced growth in soft agar, inhibition of chemotaxis and invasion. In agreement, there are several evidences demonstrating that semaphorins control the adhesion regulating integrin function in vascular morphogenesis and cancer progression [35-36]. In particular, it has been shown that Sema3A impairs endothelial cell adhesion and migration by inhibiting integrin activation [35]. In contrast but in agreement with our data, Sema3C promotes endothelial cell adhesion and migration by increasing integrin activity [36]. It is also been described that neuropilins interacting with numerous growth factors receptor and semaphorins promote tumor progression [37]. All these data suggest the complexity of Semaphorins functions and that there is much still to be learned about their involvement in tumor initiation, growth, and metastatization.

Our study demonstrated strong capability of BRAF^V600E^ clones to metastazise in mice, and in agreement with previous results [20], to form high number of big colonies in soft agar compared to NRAS^Q61R^ clones. These data were further confirmed by the strong acquired capability of NRAS^Q61R^ clones, overexpressing Sema6A, to grow in soft agar and to metastasize *in vitro* and *in vivo* suggesting a specific role of Sema6A regulating tumor progression. These finding are strongly in agreement with previous study showing that Sema6A represents, in melanoma cell line M14, one of the major modulated genes in response to MAPK inhibitor PD0325901 [38] and with our data showing that Vemurafenib treatment increases Sema6A expression in vemurafenib-resistant cells but inhibits Sema6A expression in the vemurafenib-responsive cells (unpublished data).

PlexinA2 and PlexinA4, known Sema6A receptors [39-40], showed differential expression in BRAF-mutant clones and melanomas, indicating that in such tumors only Plexin-A4 acted as Sema6A receptor. We hypothesized that depletion of Plexin-A4 may recapitulate the effects of Sema6A silencing. However, PlexinA4 silencing did not induce any of the effects of Sema6A depletion, suggesting that other, as yet unidentified, receptors could be involved. This result is in agreement with the finding that Sema6A regulates angiogenesis by a mechanism that is PlexinA2- and PlexinA4-independent [22].

The other Sema/Plexin pathway-related molecule we found highly expressed in BRAF^V600E^ cells and tumors compared to NRAS^Q61R^ cells, WT tumors and nevi, is MIical1. In vertebrates, this molecule interact with class A Plexins [41], suggesting that it could be a mediator of Sema6A signaling in melanoma cells. However, MICAL1 silencing, as well as PlexinA4, did not produce the same effects as Sema6A depletion. Specifically, we observed early and massive cell death by apoptosis in all cell lines tested suggesting that Mical1 plays a Sema6A-independent role in these tumors. These results are in agreement with the finding that Mical1 control cell survival by binding to the phosphorylation site of NDR, that in turn inhibits NDR phosphorylation-dependent apoptosis [29]. Indeed, Mical-1 depletion induced NDR phosphorylation and cell death by apoptosis.

In conclusion our results revealed that BRAF^V600E^ melanomas express high level of Sema6A and MICAL1 whose functions are strongly involved in the mechanisms that control cell proliferation and survival. Our results strongly support the hypothesis that both molecules could represent new potentially relevant therapeutic targets in BRAF^V600E^ melanoma as emerging from recent literature [41-42].

## METHODS

### Cell lines and Transfection

BRAF^V600E^ melanoma cell lines were derived from primary tumor (10538), from lymph node metastases (4686, 4023, 18732, 18656, 15392, 14464), and from s.c. 665/2 metastasis. All melanoma cell lines were isolated from surgical specimens of melanoma patients, not previously subjected to chemotherapy and admitted to Fondazione IRCCS Istituto Nazionale dei Tumori, Milan. All lesions were histologically confirmed to be cutaneous malignant melanomas [20, 43-45]. From the parental cell line 665/2 (P), which was previously shown to be a heterogeneous tumor consisting of BRAF- as well as of NRAS-mutant cells (46, 20), several BRAF^V600E^ clones (2/21, 2/33, 2/56, 2/59) or NRAS^Q61R^ clones (2/4, 2/14, 2/17, 2/51) were established as described (46). Selection of clones for the study was based on the intention to select those with the most known and relevant phenotypic differences, as previously described [20, 46-48].

The study was conducted according to the Declaration of Helsinki Principles, and following institutional guidelines. Molecular and biological features of the cell lines and clones, are described in refs [20, 43, 46]. Methods for identification of mutations in BRAF and NRAS have been previously reported [20, 43].

BRAF^V600E^ clones and primary tumor 10538, were depleted for Sem6A, Mical1, and PlexinA4 expression with specific siRNA (siSema6A, siMical1, siPlexinA4) or scramble (siScr) as described [49]. The siRNAs were either generated by Silencer® siRNA construction kit (Ambion, Glasgow, UK) or purchased from Origene or Life Technlogies.

Template oligonucleotides sequences were:

SEMA6A-5′AAATAGTCGCGGCGACTACTTCCTGTCTC3′
5′AAAAGTAGTCGCCGCGACTATCCTGTCTC3′
SEMA6A (2): AS: 5′-AAGCCTTGCTGCTATATTTCACCTGTCTC – 3′
S: 5′ – AATGAAATATAGCAGCAAGGCCCTGTCTC – 3′
SEMA6A (3): Life Technologies, Code N. AM16708
MICAL1-5′AAGGACGTCAAATTCATAGTTCCTGTCTC3′
5′AAAACTATGAATTTGACGTCCCCTGTCTC3′
MICAL1 (2):AS: 5′-AACTACTGGAGCGCCAAGTCACCTGTCTC – 3′
S: 5′-AATGACTTGGCGCTCCAGTAGCCTGTCTC – 3′
MICAL1(3): Origene, Code N. SR312119
PLEXINA4-5′AAGCAGCGGTCATTTGTCACACCTGTCTC 3′
5′AATGTGACAAATGACCGCTGCCCTGTCTC 3′
Scr1-5′AAGCGCAACTCTACCTCTACCTGTCTC3′
5′AATAGAGGTAGAGTTGCGCGCCCTG TCTC3′
Scr2-5′AATAGAGGTAGAGTTGCGCGCCCTGTCTC3′
5′AAGCGCGCAACTCTACCTCTACCTGTCTC3′

### Antibodies

Anti-Sema6A and anti-Mical1 (SIGMA-Aldrich, MO, USA), anti-total and phospho-ser473-Akt, anti-total and phospho-ERK 1/2, and anti-PARP were from Cell Signaling (Milan, Italy), anti-ErbB-2, anti-ErbB-3, anti-Caspase 3, and anti-Hsp-70 (Oncogene, MA, USA), (Santa Cruz Biotechnology, CA, USA), and (Stressgen, NY, USA), anti-phospho-NDR(Thr444) was provided by Dr. Brian Hemmings (Friedrich Miescher Institute for Biomedical Research, Basel), anti-total NDR and anti-phospho-H2B-S14, and HRP-conjugated secondary antibodies were from (Millipore, Billerica, MA, USA), and (Bio-Rad, CA, USA). For IHC, the secondary antibody was used as internal control.

### Western Blots

Clones and cell lines, before and after transfection, were lysed, analyzed by SDS-PAGE and probed (WB) with antibodies of interest and secondary HRP-conjugated antibodies [49]. Signals were detected by LuminataTM Classico Western HRP substrate (Millipore). Same amount of total protein from three independent experiments were pooled and analyzed.

### *In vivo* experiments

CD-1 male nude (nu/nu) mice, 6–8 weeks old and weighing 22-24 g were purchased from Charles River Laboratories (Calco, Italy). The procedures involving mice were in compliance with Regina Elena National Cancer Institute animal care guidelines and with national and international directives (D.L. March 4, 2014, no. 26; directive 2010/63/EU of the European parliament and of the council; Guide for the Care and Use of Laboratory Animals, United States National Research Council, 2011).

For the intracardiac experimental metastasis model, nude mice (age 8–10 weeks) were anesthetized and injected with 2×10^6^ cells of NRAS^Q61L^ (2/14, 2/17) or BRAF^V600E^ (2/21, 2/33) clones, suspended in 100 μl sterile PBS, into the left ventricle of the heart by nonsurgical means. The spontaneous, pulsatile entrance of bright red oxygenated blood into the transparent needle hub indicated proper positioning of the needle. After 5 weeks, the mice were sacrificed; selected organs were excised from the mice at necropsy and were preserved in 10% formalin solution for subsequent scoring of metastasis. NRAS^Q61L^ (2/14, 2/17) and NRAS/Sema6A cells (2/14, and 2/17) were injected intracardially as above described, and after 7 weeks the mice were sacrificed and the organs were processed and analyzed as described above. Box plot of *in vivo* metastasis analysis was obtained by software Prism version 6.0.

### Gene expression profiles

Gene expression profiles of BRAF^V600E^ and NRAS^Q61R^ melanoma clones isolated from 665/2 cell line were assessed as described [44]. Three biological replicates of each clone were analyzed. Single-color hybridization of RNAs was performed on Illumina Bead Chip HumanHT-12_v4 Microarrays (Illumina) containing more than 48,000 transcript probes. The expression profiles have been deposited in NCBI's Gene Expression Omnibus (GEO) with GSE accession number GSE58199. Background correction, filtering of data, and quantile normalization were done with BeadStudio Illumina software. Analysis for differentially expressed genes was carried out by BRB-array Tools (Vers.4.3.0) software. Class comparison was carried out by a random-variance F-test with a nominal significance level of 0.001. Permutation *P* values for significant genes were computed based on 10,000 random permutations.

### Quantitative RT-PCR

Total RNA from NRAS^Q61R^ and BRAF^V600E^ clones was prepared using TRIzol® (Ambion). First-strand cDNA was synthesized with the M-MLV RT kit (Invitrogen, Glasgow, UK). Human tissue samples were obtained from the Regina Elena National Cancer Institute, after approval by the institutional ethic committee. Total RNA, derived from nevi, BRAF^V600E^ and wild type (WT) melanoma, was isolated by PureLink FFPE kit (Invitrogen), and reverse-transcribed using PrimeScript RT reagent kit (Takara). Quantitative PCR (qPCR) was performed using SYBR Green on an ABI Prism 7500 apparatus (Applied Biosystems, Glasgow, UK) in 2 independent experiments in triplicate. The comparative threshold (∆Ct) method was used.

Primer sequences were:

SEMA6A-Fw-5′ACAATTCCTTTGTGGCACTGAA, Rev5′TCTTGAGCCGTGGAATCTGA
MICAL1-Fw-5′ATGGGCAGCCTGATGTCTCT, Rev5′GGCGCCATGCTTCTCTTG
PLEXINA4-Fw-5′TGGCTCAGGCGACCC, Rev5′GACGGAGATATTGTTGGGATG
HER3-Fw5′GCAGGATTGGTAGTGATTTTCATG,
Rev-5′TATCGCCTCATAGCCCTTTTATTC
GAPDH-Fw-5′TCCCTGAGCTGAACGGGAAG, Rev5′-GGAGGAGTGGGTGTCGCTGT

### Immunohistochemistry

Formalin-fixed paraffin-embedded sections from melanoma lymph node metastases of patients treated at the Regina Elena National Cancer Institute (53 BRAFV600E melanomas, 43 BRAF WT, and 8 nevi) were analyzed as described [42]. Antigen retrieval was performed at 96°C (10 mM/L citrate buffer, pH 6) for 20 minutes. Sections were incubated with the primary antibody anti-Mical-1 1:100 (Sigma Prestige), anti-SEMA6A 1:50 (Sigma Prestige) for 30 minutes at room temperature. Immunoreactions were revealed by Bond Polymer Refine Detection Kit according to manufacturer's procedure (Leica Biosystems) in an automated autostainer Bond III Leica Biosystems. Diaminobenzidine was used as chromogenic substrate. Microscope Nikon ECLIPSE 55i with digital camera HESP Tecnology was used. Scale bars 30 μm. The study was reviewed and approved by the ethical committee of Regina Elena National Cancer Institute, and informed consent was obtained from all patients.

### Cell adhesion, Immunofluorescence, Invasion and chemotaxis assays

siSema6A, and siScr/BRAF^V600E^ cells, 24 hours post-transfection were plated onto fibronectin (FN)-coated dishes (20 μg/ml, SIGMA-Aldrich), or culture dishes or poly-l lysine coated slides and 24 hours later analyzed by immunofluorescence. The cells incubated with Phalloidin-Tetramethylrhodamine B isothiocyanate (TRITC) were counterstained with Hoechst to highlight nuclei (SIGMA-Aldrich). For Tunel assay, siSema6A, and siScr/BRAF^V600E^ cells, 24 hours post-transfection were plated onto FN-coated or poly-l lysine coated slides, as above described. 48 hours post-transfection TUNEL assay was performed following manufacturer's instructions (Millipore). Microscope OLYMPUS BX53 was used to evaluate fluorescence and Tunel. Scale bars 20 μm. NRAS/Sema6A cells chemoinvasion was assessed as described [49] by overexpression of pUSENMC-Sema6A expression vector, kindly provided by Silvia Prislei (University Cattolica, Rome) [50]. Chemoinvasion or chemotaxis assays after depletion of Sema6A in BRAF and NRAS mutant cells, were performed as previously described [49]. Each assay was carried out in quadruplicate and repeated at least three times.

### Soft agar assay

siSema6A, siScr/BRAF^V600E^, and Sema6A/NRAS^Q61R^ cells were transfected and 48 hours later cells (5×10^3^) were plated onto 0.4% agar. After 2 weeks viable colonies were counted [42] by the software ImageJ 1.47v (NHI, USA).

### Cell death, apoptosis, and caspase activity

siMical1-, siSema6A-, and siScr/BRAF^V600E^ cells were plated at concentration of 4 × 10^5^. After 30, and 36 hours cell vitality was evaluated by Trypan blue exclusion. Apoptosis was assessed by staining with FITC-conjugated Annexin-V and Propidium Iodide (BD Pharmingen, BD Biosciences San Diego, CA). The samples were acquired by FACSCalibur flow cytometer (BD). Enzymatic activity of caspases-3/7 was measured with Muse™ Caspase-3/7 Assay Kit and read by Muse™ Cell Analyzer (Millipore).

### Statistical analysis

Data were reported as mean and standard deviation. Differences were considered statistically significant when P≤0.05. All analyses were performed using SPSS, version 17.0 (SPSS, Chicago, Illinois). Student T test was performed for the comparison of results from qRT-PCR and from all other different test (*P<0.05, **P<0.001, ***P<0.0001).

## SUPPLEMENTARY MATERIAL FIGURES AND TABLES


