# Supplementary material for: Sema6A and Mical1 control cell growth and survival of BRAFV600E human melanoma cells
Source: Oncotarget. 2014 Dec 18;6(5):2779–93. doi: 10.18632/oncotarget.2995 (PMC4413617; doi:10.18632/oncotarget.2995)
Supplement: Supplementary file 1 [file oncotarget-06-2779-s001.pdf]

# Sema6A and Mical1 control cell growth and survival of BRAF$^{V600E}$ human melanoma cells

**Supplementary Material**

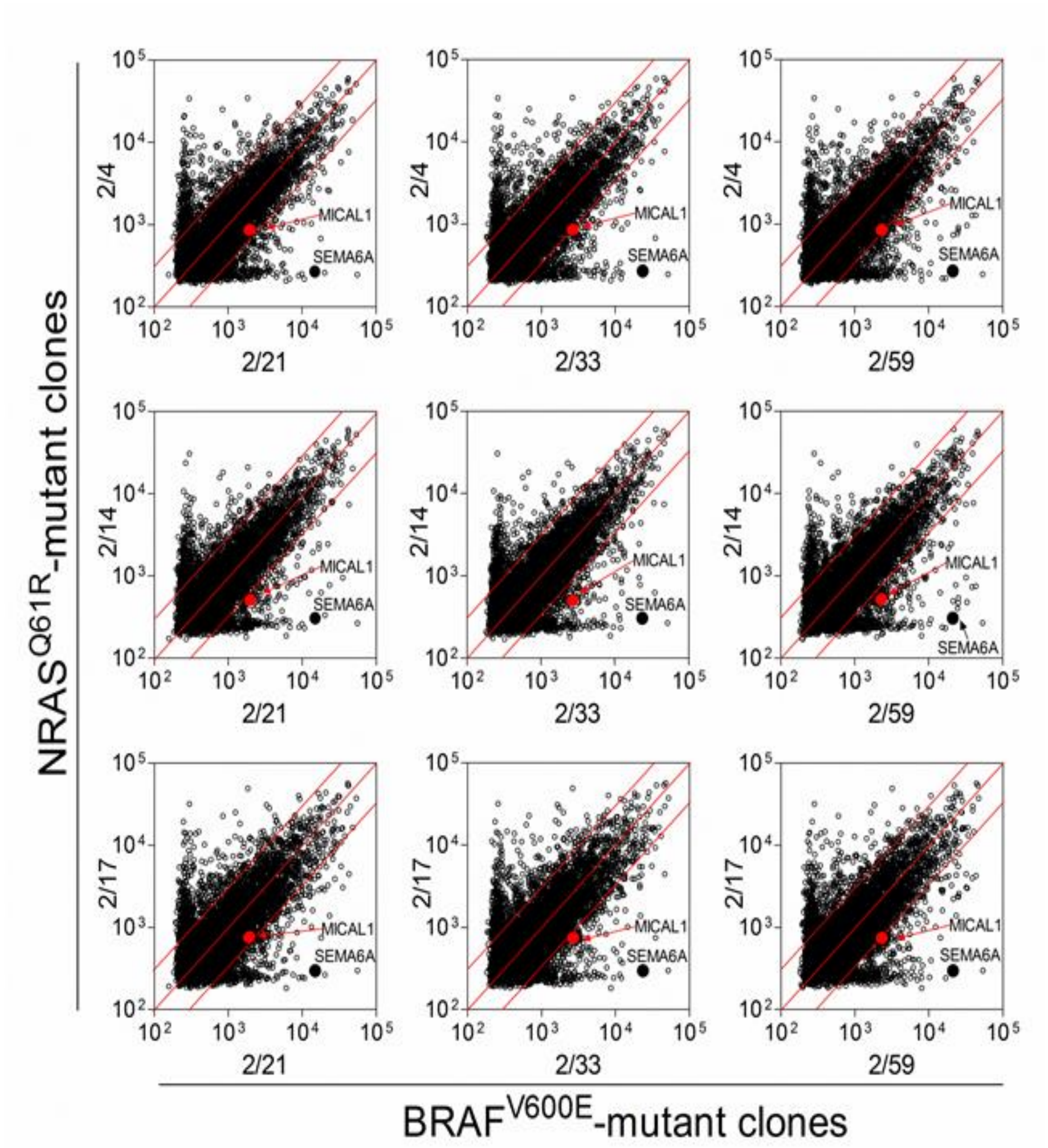


**Figure S1: Scatter plots of differentially expressed genes in BRAF-mutant and NRAS-mutant melanoma clones.** Comparison of gene expression in three BRAF-mutant melanoma clones (2/21, 2/33, 2/59) and in three NRAS-mutant melanoma clones (2/4, 2/14, 2/17) isolated from the same metastatic cell line 665/2. Whole genome gene expression profiling of the indicated clones was carried out as described in Materials and Methods. For each gene, the average expression level, based on values from three independent biological replicates, is shown. Genes located outside of the diagonal red lines have at least a three-fold difference in expression levels between BRAF-mutant and NRAS-mutant clones. Sema6A and Mical1 are identified by red symbols.

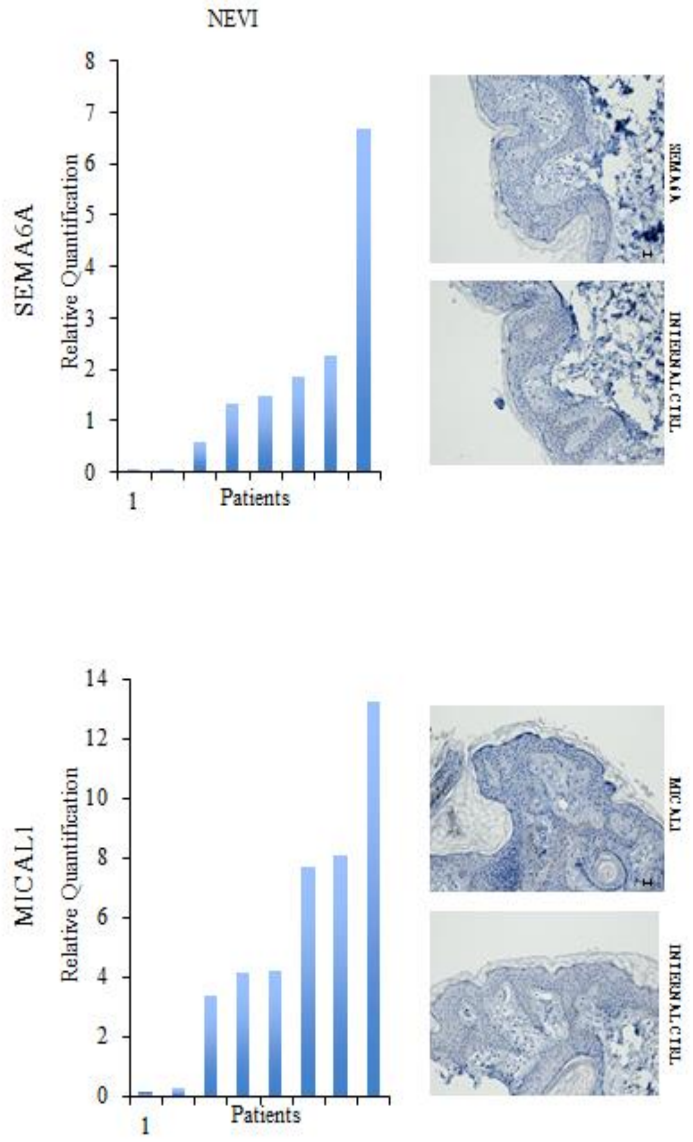


**Figure S2: Sema6A and Mical1 expression in 8 nevi.** Expression of SEMA6A and MICAL1 mRNA was examined by qRT-PCR on nevi (upper and lower graphs). Representative Sema6A and Mical1 immunohistochemistry on sections derived from same samples. Secondary antibody was used as internal control. Scale bar 30 µm.

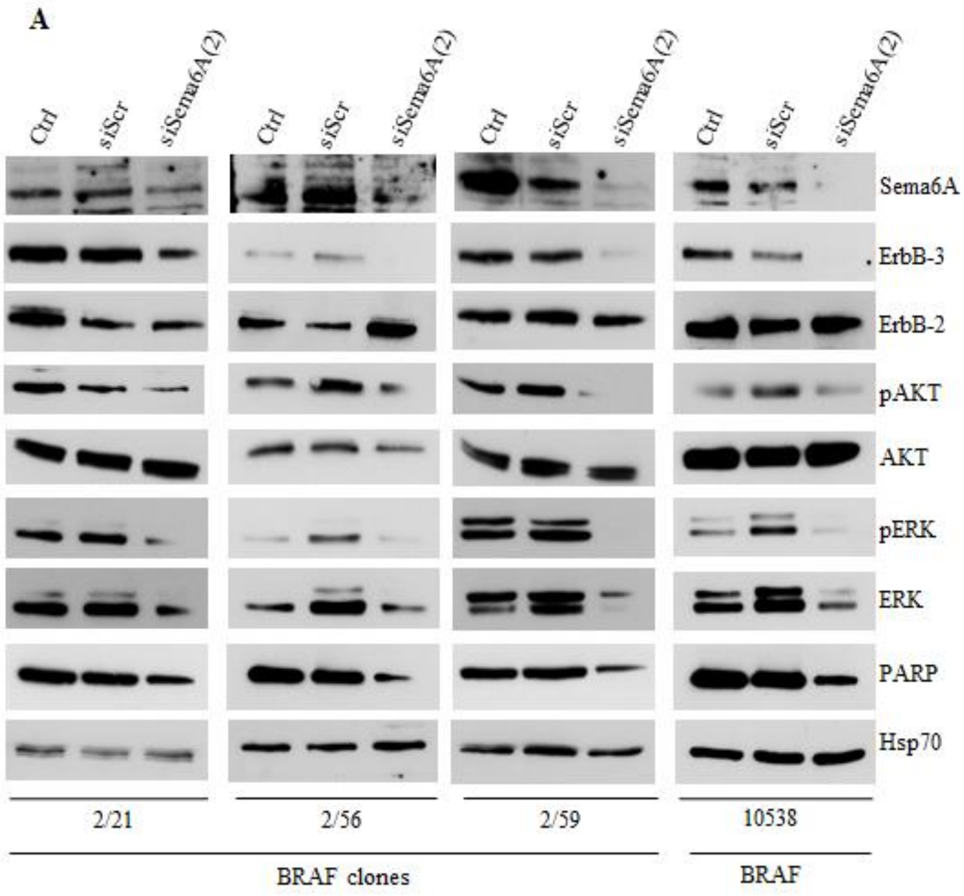


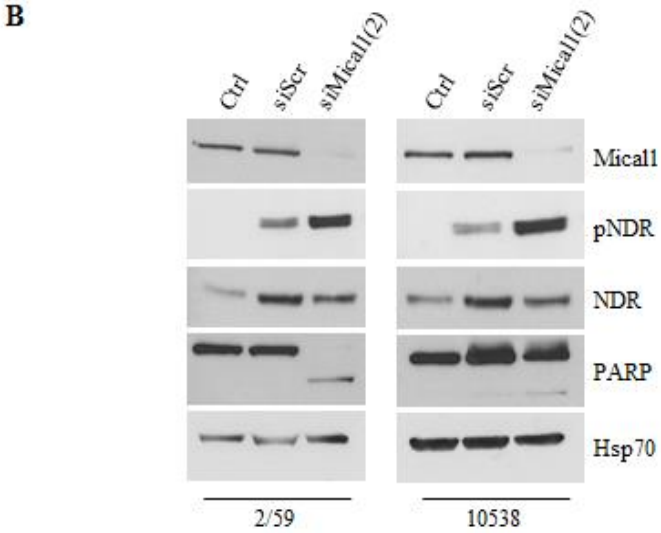


**Figure S3: Interference with a second siSema6A(2), and Mical1(2) inhibits PI3K pathway, and promotes cleavage of PARP and caspase 3**. (A) siSema6A/BRAF$^{V600E}$ clones and primary cells were analyzed by WB for expression of Sema6A, ErbB-3, ErbB-2, P-AKT, P-ERK1/2, PARP, and Hsp70. **Interference with a second siMical-1(2) induced cell death.** (B) Total cell lysate cell lysates from 2/59 and 2/56 clones, and 10538 primary tumor were analyzed at the indicated time post-transfection for expression of Mical1, total and pNDR, and PARP.

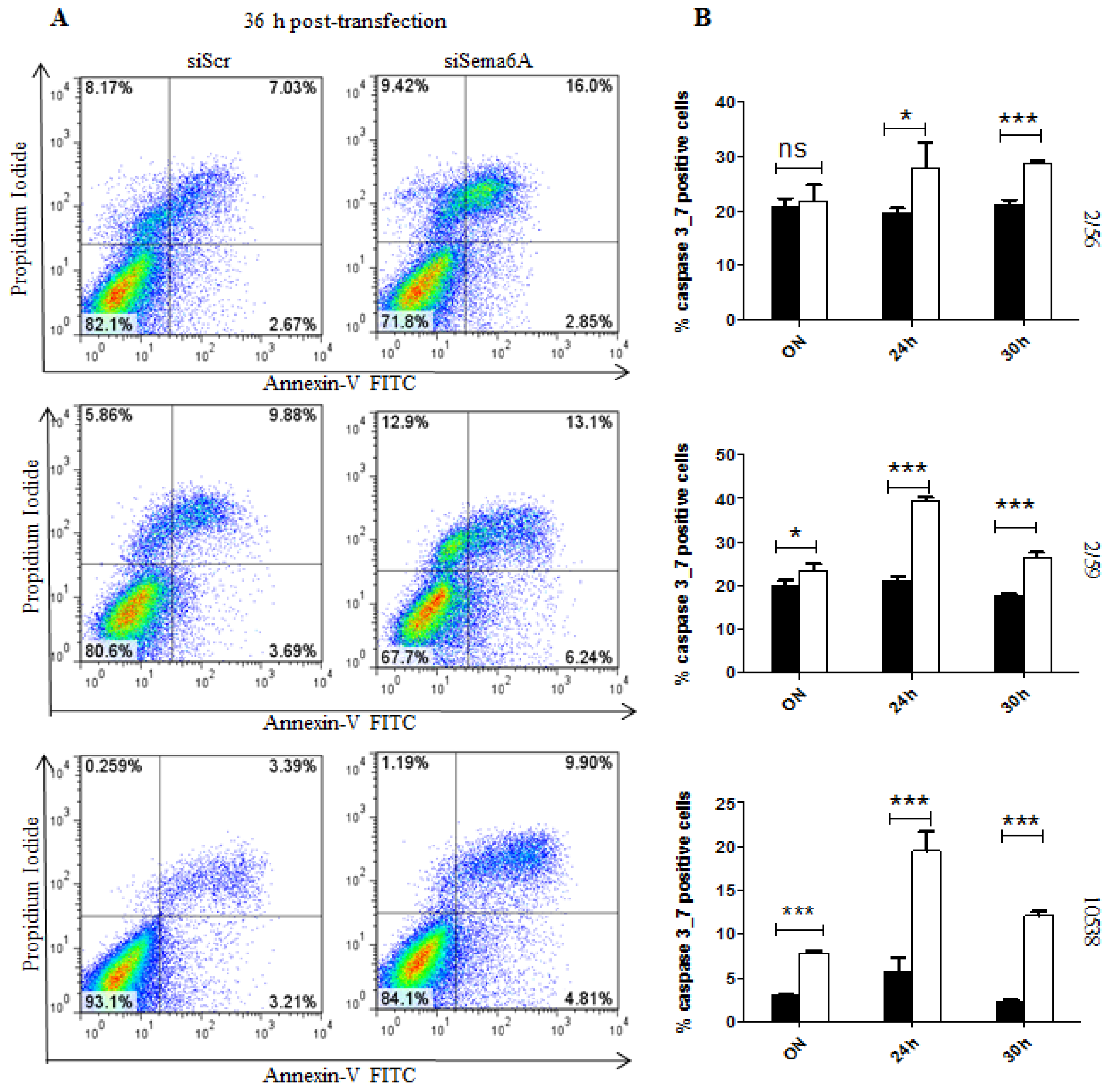


**Figure S4: Sema6A depletion induces apoptosis of BRAF$^{V600E}$ mutant melanoma**. (A) Control, siScr- or siSema6A/BRAF$^{V600E}$ clones (2/56 and 2/59) and primary BRAF$^{V600E}$ cells (10538) were analyzed with AnnexinV/PI staining (left panels) and (B) Muse™ Caspase-3/7 assay (right panels).

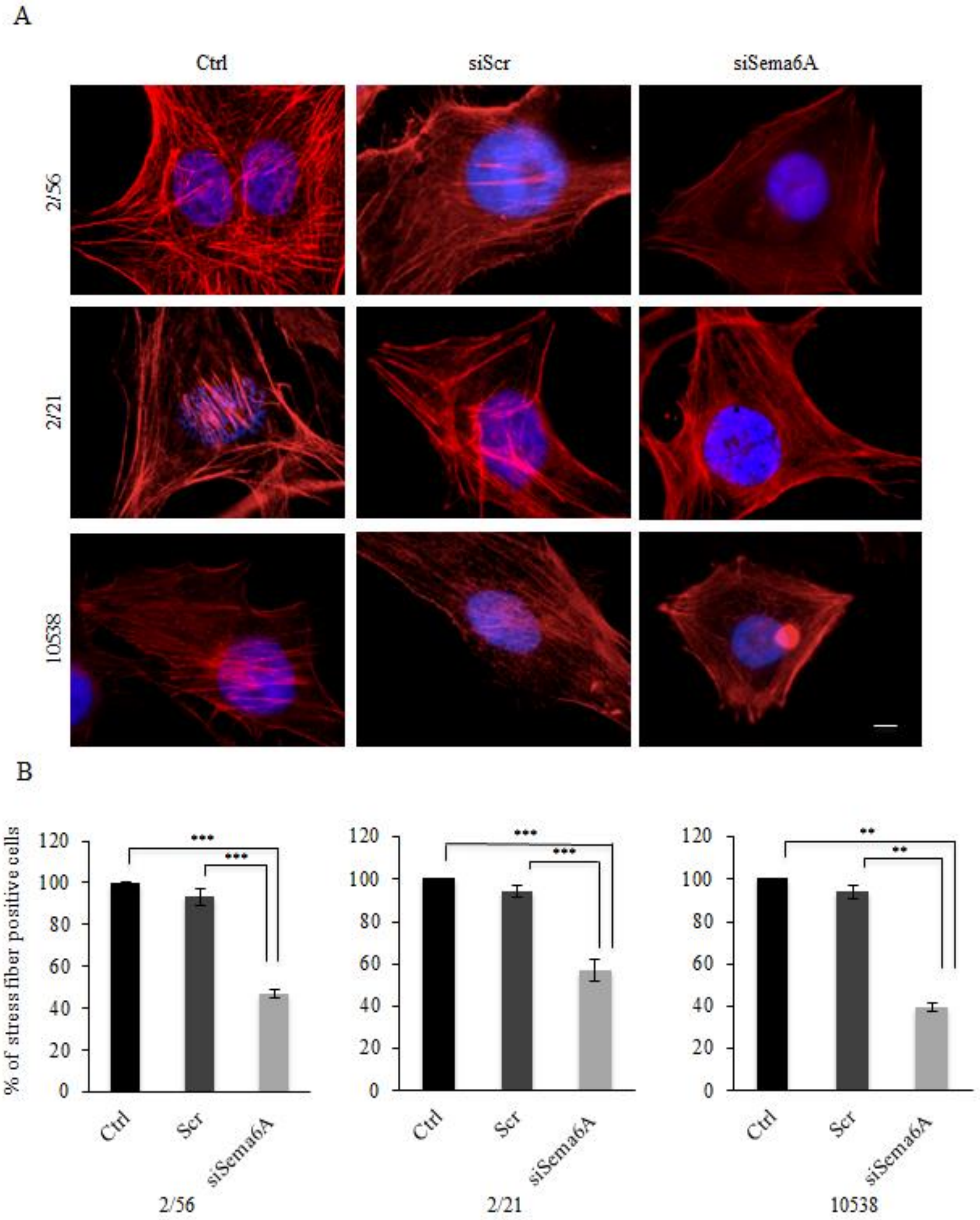


**Figure S5: Silencing of Sema6A causes disassembly of actin cytoskeleton.** (A) Ctrl, siScr- or siSema6A/BRAF$^{V600E}$ clones (2/56, 2/21), and primary cell line 10538, 24 h post-transfection, were plated on poly-l lysine coated slides. Twenty-four hours later the cells were fixed in 2% paraformaldehyde for 10 min, permeabilized and stained with phalloidin-TRITC to show actin filaments and with Hoechst to highlight nuclei and analyzed by immunofluorescence. (B) Graphical representation of cells positive to stress fibers is showed. Scale bar is 10 μm.

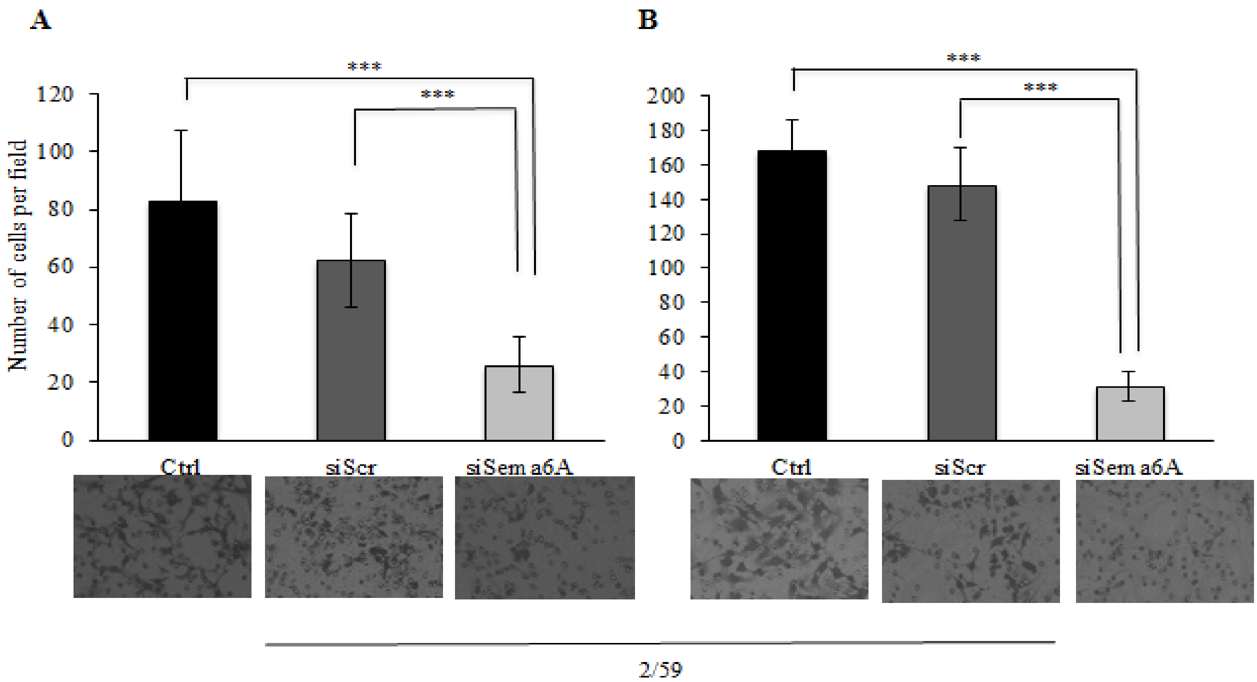


**Figure S6: Chemotaxis and chemoinvasion assays.** *In vitro* motility (A, upper and lower panels), and invasion assay (B, upper and lower panels) of BRAF/siSema6A clone 2/59. $3x10^4$ of BRAF mutant cells, after depletion of Sema6A, were assessed for their ability to migrate and invade matrigel. Each assays was carried out in quadruplicate and repeated at least three times. Statistical differences were evaluated by T test ($p<0.0001$)

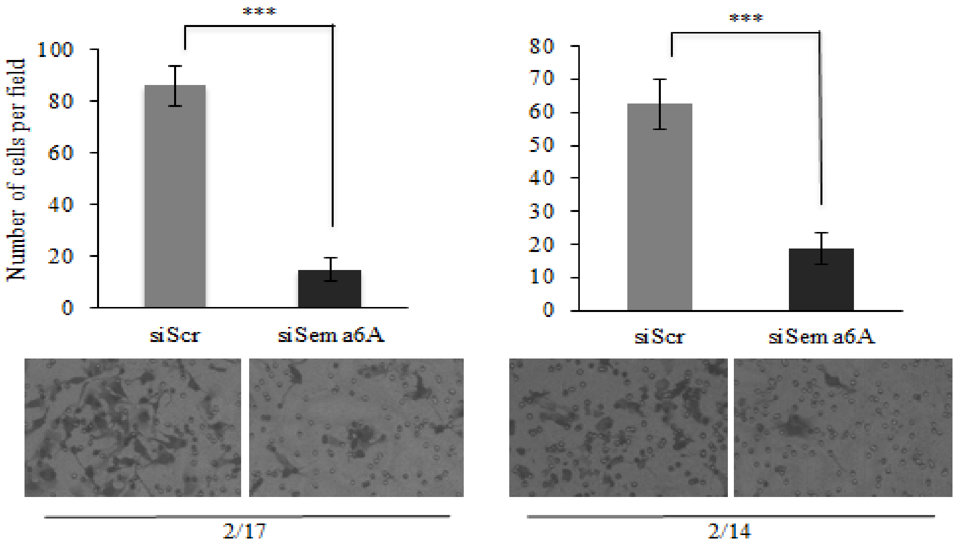


**Figure S7: Chemoinvasion assay.** Invasion assay (upper and lower panels) of NRAS/siSema6A clones 2/17 and 2/14. $3x10^4$ of NRAS mutant cells, after depletion of Sema6A, were assessed for their ability to invade matrigel. Each assays was carried out in quadruplicate and repeated at least three times. Statistical differences were evaluated by T test ($p<0.0001$).

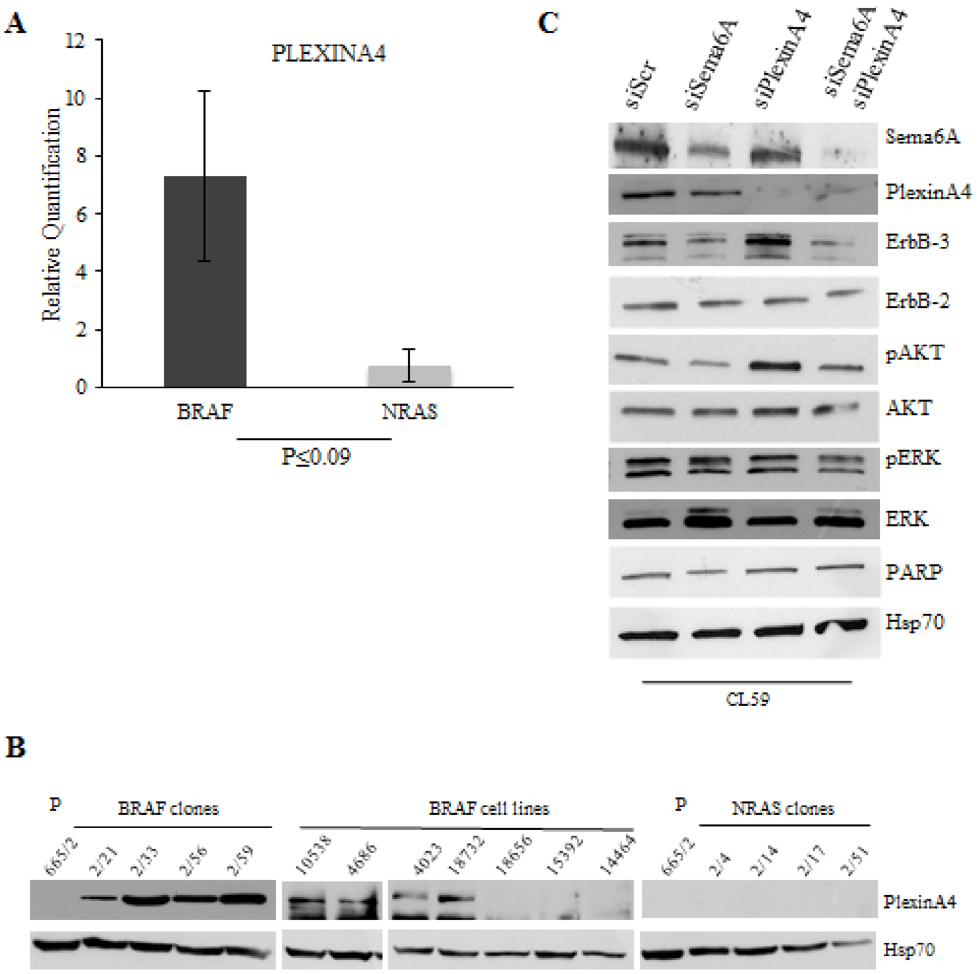


**Figure S8: RNA and protein levels of PlexinA4 in BRAF$^{V600E}$ compared to NRAS$^{Q61R}$ cells, and its function in siSema6A cells**. (A) Expression of Plexin-A4 mRNA was measured by qRT-PCR on BRAF$^{V600E}$ and NRAS$^{Q61R}$ clones; the value are shown as mean expression ± SD of 2 independent experiments in triplicate. (B) Total cell lysates from parental cell line 665/2, BRAF$^{V600E}$ clones (2/21, 2/33, 2/56, and 2/59) and cell lines (10538, 4686, 4023, 18732, 18656, 15392, 14464) or NRAS$^{Q61R}$ clones (2/4, 2/14, 2/17, and 2/51) were analyzed by WB for expression of Plexin-A4. (C) Expression of the reported protein and cell signaling analyzed by WB on Ctrl, siScr and both siSema6A/Plexin4/BRAF$^{V600E}$ on clone 2/59.

**Table S1: In vivo invasion by intracardiac injection of $BRAF^{V600E}$ and $NRAS^{Q61R}$ clones.** $2x10^6$ cells of $BRAF^{V600E}$ clones (2/21 and 2/33) and $NRAS^{Q61R}$ clones (2/14 and 2/17) were injected intracardially in four (#1-#4) and in three (#1-#3) mice, respectively. After 5 weeks, mice were sacrificed; excised selected organs were analyzed for presence (+) or not (-) of metastases. The $BRAF^{V600E}$ clones gave metastases to all organs with the exception of spleen, and in some cases more then one metastasis. Only the $NRAS^{Q61R}$ clone 2/17 was able to give two metastases, in the intestine and in the stomach respectively in one-mouse.

| MICE | | | intestine | stomach | lung | liver | brain | kidney | spleen | testis | subcutaneous |
|---|---|---|---|---|---|---|---|---|---|---|---|
| $NRAS^{Q61R}$ | 2/14 | #1 | | | | | | | | | |
| | | #2 | | | | | | | | | |
| | | #3 | | | | | | | | | |
| | 2/17 | #1 | + | + | | | | | | | |
| | | #2 | | | | | | | | | |
| | | #3 | | | | | | | | | |
| $BRAF^{V600E}$ | 2/21 | #1 | + | | | + | | | | + | + |
| | | #2 | + | + | + | | + | | | | + |
| | | #3 | | | + | | + | | | | |
| | | #4 | | | + | | | | | | |
| | 2/33 | #1 | | | | | | + | | | + |
| | | #2 | + | | + | + | + | + | | | + |
| | | #3 | | | + | | | | | | |
| | | #4 | | | + | | | | | | + |

**Table S2: In vivo invasion by intracardiac injection of $NRAS^{Q61R}$ clones overexpressing Sema6A and $NRAS^{Q61R}$ clones.** $2x10^6$ cells of $NRAS^{Q61R}$ clones (2/17 and 2/14) were injected intracardially in six mice (#1-#6); and $2x10^6$ cells of $NRAS^{Q61R}$ overexpressing Sema6A clones (2/17/Sema6A and 2/14/Sema6A) were injected in five and in four mice, respectively (#1-#5 and #1-#4); After 7 weeks, mice were sacrificed; excised selected organs were analyzed for presence (+) or not (-) of metastases. The $NRAS^{Q61R}$/Sema6A clones were highly metastatic in skeleton, lung, pancreas and heart compared to $NRAS^{Q61R}$ clones.

| MICE | | | skeleton | liver | brain | lung | pancreas | heart |
|---|---|---|---|---|---|---|---|---|
| $NRAS^{Q61R}$ | 2/17 | #1 | + | | + | | | |
| | | #2 | + | | + | | | |
| | | #3 | | | | + | | + |
| | | #4 | | | | | | |
| | | #5 | | | + | | | + |
| | | #6 | + | | | | | |
| | 2/14 | #1 | | | | + | | |
| | | #2 | | | | | | |
| | | #3 | | | | | | |
| | | #4 | | | + | | | |
| | | #5 | + | | + | | | |
| | | #6 | | | | | | |
| $NRAS^{Q61R}$/Sema6A | 2/17 Sema6A | #1 | | | | + | + | + |
| | | #2 | + | + | + | + | + | + |
| | | #3 | + | + | + | + | + | + |
| | | #4 | | | | | | |
| | | #5 | + | | | | | |
| | 2/14 Sema6A | #1 | + | | | + | | + |
| | | #2 | + | | + | + | | + |
| | | #3 | + | | | + | | + |
| | | #4 | + | | + | | | |
